# Computed tomographic evaluation of the proximity of needles placed for perineural anesthesia of the palmar digital nerves to synovial structures in the foot: an *ex vivo* study

**DOI:** 10.3389/fvets.2024.1404331

**Published:** 2024-06-04

**Authors:** Mounia Gruyaert, Maarten Oosterlinck, Maarten Haspeslagh, Annamaria Nagy

**Affiliations:** ^1^Faculty of Veterinary Medicine, Department of Large Animal Surgery, Anaesthesia and Orthopaedics, Ghent University, Merelbeke, Belgium; ^2^Equine Department and Clinic, University of Veterinary Medicine, Budapest, Hungary

**Keywords:** equine, lameness, sports medicine, orthopaedics, synovial penetration, nerve block

## Abstract

**Background:**

Potential synovial penetration following palmar digital nerve blocks has not been investigated.

**Objectives:**

To evaluate the proximity of needles placed for palmar digital nerve blocks to nearby synovial structures using computed tomography (CT).

**Study design:**

Descriptive observational study.

**Methods:**

In 18 cadaver forelimbs, sequential injection of the navicular bursa (NB), distal interphalangeal joint (DIPJ) and digital flexor tendon sheath (DFTS) was performed using 3, 5 and 10 mL diluted radiodense contrast medium, respectively. After each synovial injection, 25 gage needles were placed over the palmar digital nerves at the proximal aspect of the ungular cartilages (distal injections) and 1 cm further proximally (proximal injections), and CT examination was performed. Subsequently, needles were removed, and the synovial structures further distended with the same volume as for the first injection. Perineural needle placement and image acquisition were repeated. The distance between the needle tip and adjacent synovial structures was measured (mm) in reconstructed images. Results were analyzed in separate general linear mixed models, to determine the effect of needle position and synovial distension on the distance from the tip of the needle to the NB, DFTS and DIPJ.

**Results:**

Synovial penetration was confirmed following 12/420 (3%) needle placements (NB n = 5, 1 after proximal and 4 after distal injections; DIPJ n = 2, DFTS n = 2, NB or DIPJ n = 3, all after distal injections). The mean distance from the needle tip to the NB and DIPJ was significantly smaller after the second distension (NB: *p* = 0.025; DIPJ: *p* < 0.001) and with the distal needle placements (NB: p < 0.001; DIPJ: p < 0.001). For the DFTS, the distance from the needle tip was significantly smaller with the proximal needle placements (*p* = 0.001).

**Main limitations:**

*Ex-vivo* study.

**Conclusion:**

There is a small risk of synovial penetration when performing palmar digital nerve blocks, especially when distension of adjacent synovial structures is present.

## Introduction

Perineural anesthesia of the palmar digital nerves is frequently used to localize lameness to the distal aspect of the limb. It is performed by depositing 1–1.5 mL local anesthetic solution at, or just proximal to the proximal margins of the ungular cartilages medially and laterally, using a 25 gage 16 mm needles (1–3).

Radiodense contrast medium has been widely used to study potential post-injection distribution characteristics of local anesthetic solution (4–8). Previous studies have shown inadvertent penetration of synovial structures following perineural injection, such as the carpometacarpal joint after perineural injection of the palmar metacarpal nerves (5), the digital flexor tendon sheath after perineural injection of the palmar and palmar metacarpal lateral and medial nerves (low 4-point nerve block) (4, 9, 10) and the tarsal sheath and tarsometatarsal joint following perineural injection of the deep branch of the lateral plantar nerve (7, 8). In situations with risk of inadvertent synovial penetration, antiseptic preparation prior to performing perineural anesthesia is strongly recommended (2, 3, 11).

There is anecdotal evidence of synovial fluid appearing in the needle hub when performing perineural anesthesia of the palmar digital nerve and iatrogenic synovial infection developing shortly following palmar digital nerve anesthesia has been reported (11, 12). However, to the authors’ knowledge, there have been no published studies on the likelihood of complications following perineural anesthesia of the palmar digital nerves.

The objectives of this study were (a) to evaluate the proximity of needles placed for perineural anesthesia of the palmar digital nerves to nearby synovial structures navicular bursa (NB), distal interphalangeal joint (DIPJ), and the digital flexor tendon sheath (DFTS) using computed tomography (CT) and (b) to evaluate changes in the proximity of the needle tip with further distension of the synovial structures.

We hypothesized that inadvertent synovial penetration after perineural anesthesia of the palmar digital nerves can occur and that the needle tip would be closer to adjacent synovial structures (NB, DIPJ, and DFTS) with increased distension of the respective synovial structure.

## Materials and methods

Eighteen cadaver forelimbs (nine left and nine right) from horses euthanised for reasons unrelated to this study were used. Clinical records of the horses were unknown. The limbs had been frozen for storage and were thawed 24 h prior to injections and image acquisition. The injection sites were clipped. Sequential injection of the NB, the DIPJ and the DFTS was performed using 3, 5 and 10 mL, respectively, of 1:1 diluted contrast medium (iohexol 240 mg/mL^1^) and tap water. The volumes were based on volumes routinely used for intrasynovial anesthesia (1, 13, 14). All injections and needle placements were performed by a single operator (resident of the European College of Sports Medicine and Rehabilitation; MG). The first injection performed on each limb was either into the NB or the DIPJ. The order of first injection was alternated between the NB and DIPJ (so each structure was injected first in 50% of the limbs). The DFTS was injected after the NB and DIPJ had been injected. Following each synovial injection, two 25 gage 16 mm needles were placed subcutaneously over the palmar digital nerves on the medial and lateral side. The needles were inserted just proximal to the palpable proximal edge of the medial and lateral ungular cartilages and were directed distally (1). A second needle was placed on both the medial and lateral sides, 1 cm proximal to the first insertion sites, also pointing distally. This was done mimicking a more proximal needle placements executed by some clinicians (14, 15). Following each synovial injection and perineural needle placement, a CT examination was performed. The needles over the palmar digital nerves were kept *in situ* for each CT examination, but were removed prior to any further synovial injection. When the subsequent synovial injection was deemed successful (based on synovial fluid appearing in the needle hub and/or contrast fluid being easily injected without any resistance), two 25 gage 16 mm needles were placed again as described above. The intrasynovial needles were kept *in situ* to allow subsequent injections (see later). To prevent leakage, a cap was attached to the needle hub. Subsequently, the same steps were repeated; each synovial structure was distended further with the previously used volume to mimic marked synovial distension. For the NB, a 19 gage 88 mm spinal needle was inserted in the midline between the heel bulbs immediately proximal to the coronary band, aiming halfway between the most dorsal and the most palmar aspects of the coronary band and 1 cm distal to the coronary band (1, 16). Correct needle placement was confirmed by a lateromedial radiograph. For the DIPJ injection, a 20 gage 38 mm needle was inserted perpendicular to the skin, 1 cm proximal to the coronary band, into the dorsal pouch of the DIPJ. The DFTS was injected using a 20 gage 38 mm needle, inserted at the axial border of the lateral proximal sesamoid bone (17). A CT examination was performed after each distension; each limb was scanned six times in total. A 16 slice multidetector fan beam CT (2) (Qalibra CT System, Canon Aquilion LB) was used. The images were acquired with a slice thickness of 0.5 mm (tube rotation time 0.5 s). The field of view was 320 mm and the images were generated at 350 mAs and 135 kV.

**Figure 1:**
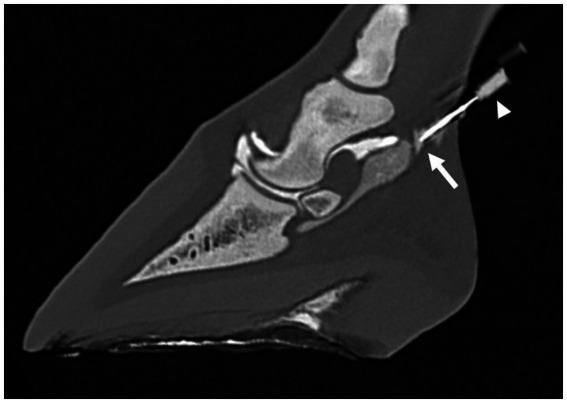
Parasagittal computed tomographic reconstruction, showing penetration of the navicular bursa (arrow) by a needle inserted just proximal to the ungular cartilage after the second distension of the navicular bursa with 3 mL of diluted contrast medium. Contrast leakage from the needle hub is clearly visible (arrowhead).

**Figure 2:**
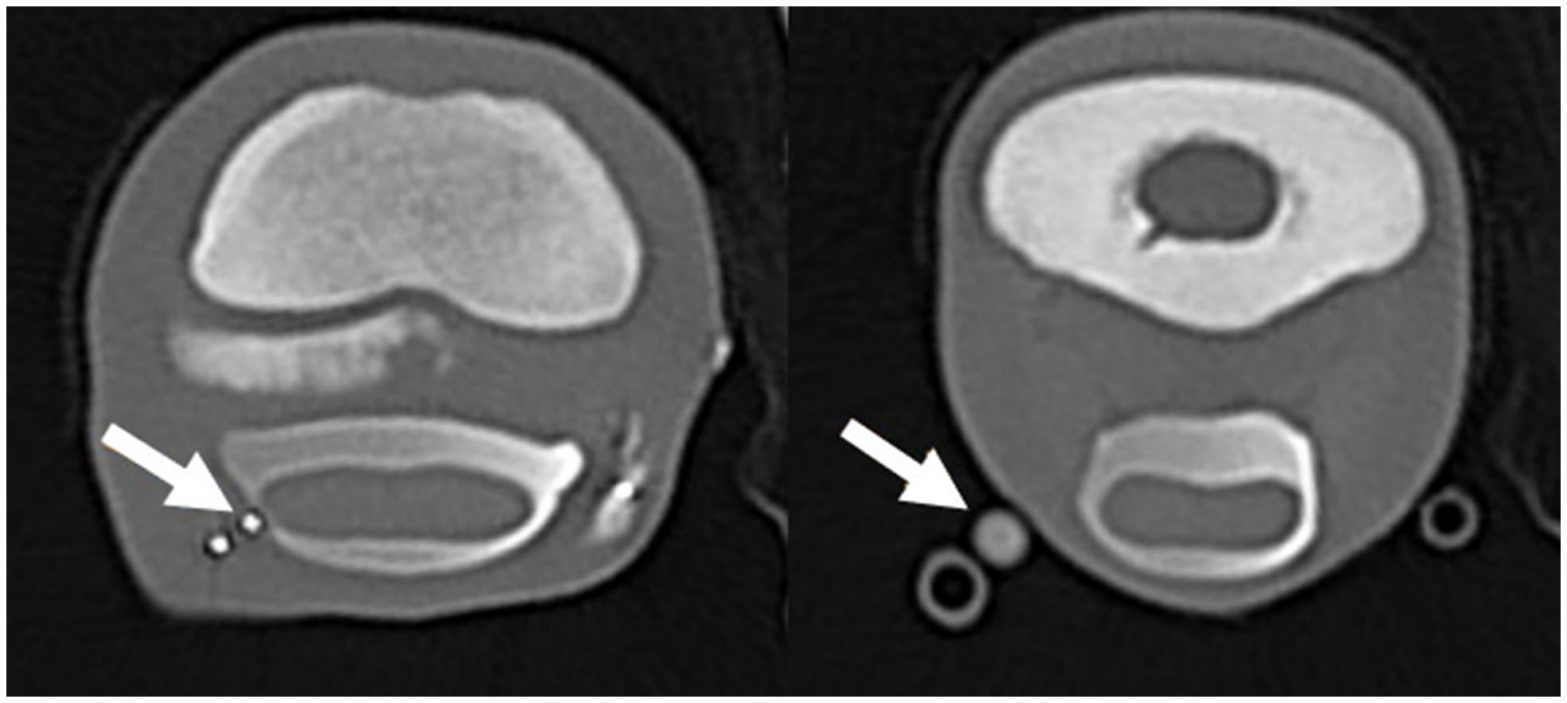
Transverse computed tomographic reconstruction, showing penetration of the digital flexor tendon sheath (DFTS) (arrow left image) and contrast leakage from the proximal (medial) needle (arrow right image) after the second distension with 10 mL diluted radiodense contrast medium. The arrow on the left image is showing the distal needle tip penetrating the DFTS; the right image is showing contrast leakage from the needle hub.

**Figure 3:**
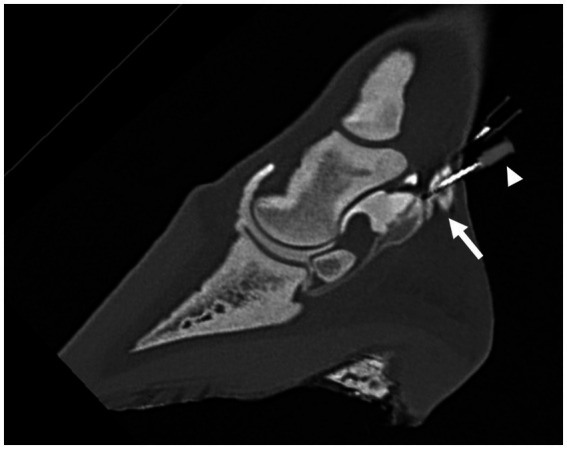
Parasagittal computed tomographic reconstruction, showing penetration (arrow) of either the navicular bursa or the distal interphalangeal joint by the distal needle after the second distension of the NB and DIPJ with 3 mL and 5 mL of diluted radiodense contrast medium, respectively. It was not possible to differentiate if the needle penetrated the NB or the DIPJ. Contrast leakage from the needle hub is visible (arrowhead).

**Figure 4:**
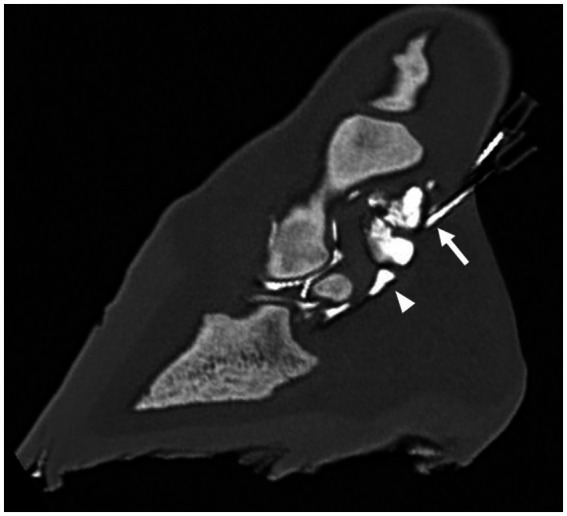
Parasagittal computed tomographic reconstruction, showing the distal needle adjacent to the distal interphalangeal joint (arrow) after a single distension with 5 mL diluted radiodense contrast medium. Note that the navicular bursa (arrowhead) has also been distended with contrast medium.

Images were analyzed using multiplanar reconstruction (MPR) and a bone algorithm in a medical image viewing software [JiveX (3)]. After assessment of different reconstructions, it was decided that the most suitable plane to measure the shortest distance between the tip of the needle and the synovial structures was the sagittal plane for the NB and DIP joint and the transverse plane for DFTS. In the sagittal plane, the reference lines were set parallel with the deep digital flexor tendon and perpendicular to this line. In the transverse plane, reference lines were set parallel with the palmar surface of the navicular bone and perpendicular to this line. All measurements were performed three times, and the shortest distance (mm) from the tip of the needle to the injected synovial structure was used for further analysis. Five categories were defined: 1. Penetration: inadvertent penetration of a synovial structure, confirmed by presence of contrast medium in the needle hub (Figures 1–3); 2. Adjacent: the needle tip was adjacent to the synovial structure but no contrast leakage was noted (Figure 4); 3. The needle tip was not adjacent but <5 mm from the synovial structure; 4. a distance of ≥5 mm but <10 mm between the needle tip and the synovial structure and 5. a distance of ≥10 mm between the needle tip and the synovial structure.

Two limbs with major tendon abnormalities (rupture of both superficial digital flexor tendon and deep digital flexor tendon) observed during CT evaluation were excluded from the study.

### Data analysis

Statistical analysis was carried out in IBM SPSS Statistics 26.0 (4). Statistical significance was set at *p* ≤ 0.05. Descriptive statistics were performed in spreadsheet software (Microsoft Excel version 16). To determine the effect of needle position and synovial distension on the distance from the tip of the needle to the NB, DFTS and DIPJ, a separate general linear mixed model was used for each synovial structure, with distance from the needle tip to the synovial structure as dependent variable, distension (first/s), needle position (proximal/distal) and their interaction as fixed effects, and limb and location within limb (lateral/medial) as random effects. Normality of residuals for these models was visually verified on QQ-plots and formally confirmed with a Kolmogorov–Smirnov test. If residuals could not be assumed to be normally distributed, the analysis was performed using generalized estimating equations with identity link function on rank-transformed data instead, with the lowest distance yielding the lowest rank (18). In the latter case, the presence of related measurements in the dataset was addressed by including limb and location within limb (lateral/medial) in the model as subject effects in an unstructured correlation matrix.

## Results

In total, there were 420 needle placements over the palmar digital nerves, of which 204 were at the proximal and 216 at the distal injection site (in the first limb, proximal injections were not performed). Synovial penetration was confirmed following 12/420 (3%; 95% confidence interval [CI] 1.5–4.9%) needle placements (Table 1). In 11/12 (92%; 95% CI 61.5–99.8%) needle placements, this occurred after the second distension. Of the 12 synovial penetrations, 10 (83%; 95% CI 51.2–98.0%) occurred following distal needle placements.

**Table 1:** The number of penetrations of the navicular bursa (NB), distal interphalangeal joint (DIPJ), and digital flexor tendon sheath (DFTS) following perineural needle placement over the palmar digital nerves.

	First distension		Second distension		TOTAL penetrations
	Proximal	Distal	Proximal	Distal	N
NB	0	1	0	4	5
DIPJ	0	0	0	2	2
NB/DIPJ	0	0	0	3	3
DFTS	0	0	2	0	2
TOTAL	0	1	2	9	12

The synovial structures were injected with 3 mL (NB), 5 mL (DIP), and 10 mL (DFTS) of 1:1 diluted contrast medium. After each injection (“First Distension”) needles were inserted just proximal to the palpable edge of the medial and lateral ungular cartilages and were directed distally (“Distal”). The second needles were placed 1 cm proximal to the first insertion sites (“Proximal”). Subsequently, the same steps were repeated; each synovial structure was injected a second time with 3 mL (NB), 5 mL (DIP) and 10 mL (DFTS) of 1:1 diluted contrast medium to mimic marked synovial distension (“Second Distension”).

Following 11/420 (3%; 95% CI 1.3–4.6%) needle placements, the tip of the needle was adjacent to a synovial structure, but no contrast leakage was seen (Supplementary item 1). In 7/11 limbs (64%), this was after the second distension (DIPJ *n* = 5, DFTS *n* = 6) and in 7/11 limbs (64%) with the proximal needle placement (DFTS *n* = 6, DIPJ *n* = 1, DIPJ *n* = 4). In one limb, contrast was noted in the DIPJ after injection of the NB, indicating direct communication between the two structures. The results of the other categories are shown in Supplementary item 1.

### Statistical analysis

#### Navicular bursa

The mean distance from the distal needle tip to the NB was significantly smaller (*p* = 0.025) after the second than after the first distension (Table 2). The mean distance from the needle tip to the NB was significantly smaller with the distal than with the proximal needle placements (*p* < 0.001). Four of five penetrations of the NB occurred after the second distension and all five were with distal needle placements (Table 1).

**Table 2:** Distance from the distal needle tip to the navicular bursa (NB), distal interphalangeal joint (DIPJ), and digital flexor tendon sheath (DFTS).

	First Distension (mm)	Second Distension (mm)	*p*-value	Proximal needle placement (mm)	Distal needle placement (mm)	*p*-value
NB (mean ± sd)	18.5 ± 8.6	17.5 ± 8.8	0.025*	21.7 ± 7.8	14.4 ± 8.0	<0.001*
DIPJ (mean ± sd)	11.7 ± 5.3	9.1 ± 6.0	<0.001*	13.4 ± 5.5	7.5 ± 4.4	<0.001*
DFTS (median and range)	2.6 (0–14)	2.0 (0–12)	0.5	1.7 (0–6.1)	3.0 (0–13.9)	0.001*

Normally distributed data are shown as mean ± standard deviation (NB and DIPJ), whereas data not normally distributed are presented as median and range (DFTS). The synovial structures were injected with 3 mL (NB), 5 mL (DIP) and 10 mL (DFTS) of 1:1 diluted contrast medium. After each injection (“First Distension”) needles were inserted just proximal to the palpable proximal edge of the medial and lateral ungular cartilages and were directed distally (“Distal”). The second needles were placed 1 cm proximal to the first insertion sites (“Proximal”). Subsequently, the same steps were repeated; each synovial structure was injected a second time with 3 mL (NB), 5 mL (DIP) and 10 mL (DFTS) of 1:1 diluted contrast medium to mimic marked synovial distension (“Second Distension”). *p*-values illustrate statistically significant (*p* ≤ 0.05) differences between first and second distensions, and between proximal and distal needle placements.

#### Distal interphalangeal joint

The mean distance from the needle tip to the DIPJ was significantly smaller (*p* < 0.001) after the second than after the first distension. The mean distance from the needle tip to the DIPJ was significantly smaller with the distal than with proximal needle placements (*p* < 0.001) (Table 2).

Following 115/140 (82.1%) needle placements, the distance from the needle tip was <5 mm away from the DIPJ. Six of seven penetrations and adjacent needle placements occurred with the distal needle placements (Supplementary item 1).

#### Digital flexor tendon sheath

No significant difference was noted between the median distance from the needle tip to the DFTS between first and the second distension. The distance from the needle tip the to the DFTS was significantly shorter with the proximal than with distalneedle placements (*p* = 0.001) (Table 2).

Following 98/140 (70.0%) needle placements, the distance from the needle tip was <5 mm from the DFTS. Six of eight penetrations and adjacent needle placements occurred after the second distension, all of which with the proximal needle placements (Supplementary item 1).

## Discussion

This study is the first to perform a detailed evaluation of the proximity of needles placed for perineural anesthesia of the palmar digital nerves to synovial structures in the foot. In this study, we focused on the NB, DIPJ and DFTS because of their close relationship to the injection sites of perineural anesthesia of the palmar digital nerves. The NB, the palmaroproximal pouch of the DIPJ and the distal aspect of the DFTS are closely related, separated by the proximal sesamoidean ligament (also called T-ligament or transverse laminae) and the collateral sesamoidean ligament (19–21). The proximal sesamoidean ligament is loose connective tissue corresponding to the apposition of palmaroproximal recess of the DIPJ, the proximal recess of the NB and distal recess of the DFTS and lies in close relationship to the collateral sesamoidean ligament which originates on the medial and lateral aspect of the proximal phalanx and inserts on the proximal aspect of the navicular bone (21, 22). The proximal interphalangeal joint lies dorsal to the deep digital flexor tendon (and DFTS) and was therefore not investigated in this study (23).

In agreement with our hypotheses, synovial penetration occurred only after a small proportion of injections, and the distance from the needle tip to adjacent synovial structures was significantly smaller when the NB and the DIPJ had been distended twice. Also, the distance from the needle tip to adjacent synovial structures (NB and DIPJ) was significant smaller with the distal needle placements. The latter finding can be explained by the anatomical location of the NB (distal to the proximal ungular cartilages) and of the palmaroproximal pouch of the DIPJ. In contrast, the distance from the needle tip to the DFTS was significant smaller with proximal needle placements. This could be explained by the presence of the additional soft tissue coverage by the distal digital annular ligament in this region, which is not present more proximally (23).

Our results suggest that there is a small risk of synovial penetration when performing perineural anesthesia of the palmar digital nerves, at least for the NB and the DIPJ. As the perineural injections are performed near the proximal margin of the ungular cartilages, the direction and location of the needle placement in relation to the ungular cartilages could play a role (3, 24). In practice, some variations in execution of the perineural anesthesia of the palmar digital nerves among veterinarians exist. A first factor is the location of the needle placement in relation to the ungular cartilages. A slightly more proximal injection site in relation to the ungular cartilages can be used, but this increases the risk of proximal diffusion and potential desensitization of the pastern and distal fetlock region (16, 25). A second factor is the direction of the needle. In the current study, and as described in most reference texts, the needles were inserted subcutaneously in a proximal to distal direction, which results in the needle tip ending distally to the skin penetration site (1–3). For the NB and DIPJ, more penetrations occurred with the distal needle placements. As discussed earlier, this finding can be explained by the anatomical location of the NB and DIPJ. It could therefore be considered to direct the needle perpendicular to the skin, to avoid a more distal position of the needle tip. In combination with the use of a shorter 26 gage, 13 mm needle, it can be speculated that the distance to the nearby synovial structures could be decreased and therefore, the risk of inadvertent synovial penetration could be mitigated. Two of the authors routinely use this modification (26 gage, 13 mm needles in combination with needle insertion perpendicular to the skin) for perineural anesthesia of the distal digital nerves. However, for cob types and other horses with a thick skin, a 16 mm and ≤ 26 gage needles may be necessary. Further studies need to be performed to assess the effect of these modifications on the resulting distance of the needle tip to the adjacent synovial structures.

If synovial penetration occurs while performing perineural anesthesia of the palmar digital nerves, it is possible that the loss of local anesthetic solution into a synovial structure results in an incomplete desensitization of the nerve. Inadequate nerve desensitization can be detected by checking loss of skin sensitivity at the heel bulbs, although there is not a complete correlation between loss of skin sensitivity and resolution of lameness due to foot pain (2, 26).

Several previous *(ex vivo)* studies have shown potential inadvertent penetration of synovial structures such as the carpometacarpal joint, the DFTS, the tarsal sheath and tarsometatarsal joint following perineural injections (4, 5, 7–10). Two of these studies (7, 9) have investigated using different volumes at the injection sites, but no studies have distended the synovial structures prior to the perineural injection. In our study, penetration of a synovial structures occurred in 12/420 (3%) of needle placements and more frequently after the second distension, suggesting that inadvertent synovial penetration is more likely if adjacent synovial structures are markedly distended, at least for the NB and the DIPJ. Although based on published scientific literature and the authors’ clinical experience, iatrogenic infections after performing perineural anesthesia in general, and of the palmar digital nerves specifically, are very rare, clinicians should be aware of the potential risk of inadvertent synovial penetration. Based on our study, this may be particularly relevant when there is a palpable distension of adjacent synovial structures. Therefore, thorough palpation of synovial structures should always be performed, although this is not possible for the NB due to its anatomical location. Theoretically, this could be visualized by ultrasonographic evaluation but this is not a practical approach prior to perineural anesthesia in a clinical setting. A potential explanation for the low incidence of iatrogenic synovial infection after perineural anesthesia may be that not every synovial penetration would necessarily result in synovial contamination and infection. Local anesthestics present antimicrobial activity against equine bacterial pathogens at concentrations that are used in practice (27).

In one limb, diffusion of the contrast medium from the NB to the DIPJ was noted. In one earlier study using CT arthrography, an occasional direct communication from the DIPJ to the NB was reported in 7/133 (5.3%) cadaver limbs (13). The authors stated that communication could occur through the proximal sesamoidean ligament or the distal sesamoidean impar ligament. The latter could be associated with the presence of a distal border fragment (13). This can be an important consideration in the context of intrasynovial anesthesia of the NB or DIPJ but is not directly relevant to our study.

This study had some limitations. This is an *ex-vivo* study on a relatively small number of limbs, and the distension induced during the first and second synovial injections might not reflect naturally occurring synovial distension. Any baseline distension prior to injection of contrast medium was not assessed. However, the experimental design of the study allowed studying the effect of a standardized mild and marked synovial distension, in combination with very detailed evaluation using cross-sectional CT imaging. Despite a single experienced operator performing all procedures using the same technique, a slight variation in the location and orientation of perineural needle placements between distensions might have occurred. The clinical history of the horses was not available but if major abnormalities were observed during the CT evaluation, limbs were excluded, and therefore, this is considered unlikely to have affected our results.

Further studies to better assess the potential risk of synovial penetration in clinical situations could include *in-vivo* studies by performing an injection with local anesthetic solution and/or radiodense contrast medium over the palmar digital nerves, followed by radiographic assessment of any contrast accumulation in nearby synovial structures (4, 5, 15). Alternatively, yet more complicated, it could be considered to measure the concentration of local anesthetic solution in nearby synovial structures (14).

In conclusion, inadvertent penetration of the DIPJ, NB or DFTS may occur when performing perineural anesthesia of the palmar digital nerves, although based on this *ex vivo* study, the risk seems very low. Nevertheless, clinicians should be aware of this potential risk and needle size and direction may warrant further consideration and research.

## Data availability statement

The raw data supporting the conclusions of this article will be made available by the authors, without undue reservation.

## Ethics statement

Ethical approval was not required for the studies involving animals in accordance with the local legislation and institutional requirements because the material used was derived from horses that were subject to euthanasia for unrelated reasons. Written informed consent was not obtained from the owners for the participation of their animals in this study because by signing the general consent form of the hospital on admission of all cases, owners agree for data acquired on their horses to be used for teaching and research purposes.

## Author contributions

MG: Conceptualization, Investigation, Methodology, Validation, Writing – original draft, Writing – review & editing. MO: Conceptualization, Funding acquisition, Investigation, Methodology, Supervision, Validation, Writing – original draft, Writing – review & editing. MH: Data curation, Formal analysis, Writing – original draft, Writing – review & editing. AN: Conceptualization, Funding acquisition, Investigation, Methodology, Supervision, Validation, Writing – original draft, Writing – review & editing.

## References

[1] Schumacher J Schramme MC DeGraves FJ Smith R Coker M. Diagnostic analgesia of the equine forefoot. Eq Vet Educ. (2004) 16:159–65. doi: 10.1111/j.2042-3292.2004.tb00288.x

[2] Bassage LH II Ross MW. Chapter 10: diagnostic analgesia In: Ross MW Dyson SJ, editors. Lameness in the horse. 2nd ed. St. Louis, MO: Saunders (2011)

[3] Baxter GM Stashak TS. Chapter 2: examination for lameness In: Baxter GM, editor. Lameness in horses. 7th ed. Hoboken, NY: Wiley Blackwell (2020)

[4] Nagy A Bodo G Dyson S Compostella F Barr ARS. Distribution of radiodense contrast medium after perineural injection of the palmar and palmar metacarpal nerves (low-4-point nerve block: an *in vivo* and *ex vivo* study in horses). Equine Vet J. (2010) 42:512–8. doi: 10.1111/j.2042-3306.2010.00076.x, PMID: 20716191

[5] Nagy A Bodo G Dyson S. Diffusion of contrast medium after four different injection techniques for analgesia of the proximal metacarpal region: an *in vivo* and *in vitro* study. Equine Vet J. (2012) 44:668–73. doi: 10.1111/j.2042-3306.2012.00564.x, PMID: 22471337

[6] Jordana M Marten A Duchateau L Vanderperren K Saunders J Oosterlinck M. Distal limb desensitisation following analgesia of the digital flexor tendon sheath in horses using four different techniques. Equine Vet J. (2013) 46:488–93. doi: 10.1111/evj.12186 24033590

[7] Claunch KM Eggleston RB Baxter GM. Effects of approach and injection volume on diffusion of mepivacaine hydrochloride during local analgesia of the deep branch of the lateral plantar nerve in horse. J Am Vet Med Assoc. (2014) 245:1153–9. doi: 10.2460/javma.245.10.1153, PMID: 25356717

[8] Contino EK King MR Valdés-Martinez A McIIWraight. In vivo diffusion characteristics following perineural injection of the deep branch of the lateral plantar nerve with mepivacaine or iohexol in horses. Equine Vet J. (2015) 47:230–4. doi: 10.1111/evj.12261, PMID: 24612216

[9] Seabaugh KA Selberg KT Valdés-Martinez A Rae S Baxter GM. Assessment of the tissue diffusion of anesthetic agent following administration of a low palmar nerve block in horses. J Am Vet Med Assoc. (2011) 239:1334–40. doi: 10.2460/javma.239.10.1334, PMID: 22044331

[10] de Souza AF Pascual JC Maia BT De Zoppa AL. Diffusion of dye after perineural injection of the palmar/plantar nerves in two different sites in horses: an ex-vivo study. Vet Res Commun. (2022) 46:283–8. doi: 10.1007/s11259-021-09856-6, PMID: 34713307

[11] Schumacher J Schramme MC Schumacher J DeGraves FJ. Diagnostic analgesia of the equine digit. Equ Vet Educ. (2013) 25:408–21. doi: 10.1111/eve.12001

[12] Pilsworth R Dyson S. Where does it hurt? Problems with interpretation of regional and intra-synovial diagnostic analgesia. Eq. Vet. Edu. (2015) 27:595–603. doi: 10.1111/eve.12392

[13] Hontoir F Rejas E Falticeanu A Nisolle J-F Simon V Nicaise C. Communication between the distal interphalangeal joint and the navicular bursa in the horse at computed tomography arthrography. Anat Histol Embryol. (2019) 48:133–41. doi: 10.1111/ahe.12421, PMID: 30609106

[14] Gough M Mayhew IG Munroe GA. Diffusion of mepivacaine between adjacent synovial structures in the horse. Part 1: forelimb foot and carpus. Equine Vet J. (2002) 34:80–4. doi: 10.2746/042516402776181097, PMID: 11817556

[15] Schumacher J Livesey L Degraves FJ Schumacher J Schramme MC Hatchcock J. Effect of anaesthesia of the palmar digital nerves on proximal interphalangeal joint pain in the horse. Equine Vet J. (2004) 36:409–14. doi: 10.2746/0425164044868404, PMID: 15253081

[16] Nagy A Malton R. Diffusion of radiodense contrast medium after perineural injection of the palmar digital nerves. Eq Vet Educ. (2015) 27:648–54. doi: 10.1111/eve.12369

[17] Verschooten F Desmet P Peremans K Picavet T. Navicular disease in the horse: the effect of controlled intrabursal corticosteroid injection. J Equine Vet. (1991) 11:316–20.

[18] Hassel DM Stover SM Yarbrough TB Drake CM Taylor KT. Palmar-plantar axial sesamoidean approach to the digital flexor tendon sheath in horses. J Am Med Vet. (2000) 217:1343–7. doi: 10.2460/javma.2000.217.1343, PMID: 11061387

[19] Fan C Zhang D. Wald-type rank test: a GEE approach. Comput Stat Data Anal. (2014) 74:1–16. doi: 10.1016/j.csda.2013.12.004

[20] Bowker RM Linder K Van Wulfen KK Sonea IM. Anatomy of the distal interphalangeal joint of the mature horse: relationships with navicular suspensory ligaments, sensory nerves and neurovascular bundle. Equine Vet J. (1997) 29:126–35. doi: 10.1111/j.2042-3306.1997.tb01654.x, PMID: 9104562

[21] Hontoir F Paques F Simon V Balau B Nicaise C Clegg P. Is the T-ligament a ligament? A histological study in equine cadaver forelimbs. Res Vet Sci. (2020) 132:10–6. doi: 10.1016/j.rvsc.2020.05.018, PMID: 32470846

[22] Butcher MT Bertram JEA Benzuidenhout AJ. Collateral ligaments of the distal sesmoid bone in the digit of Equus: re-evaluating midstance function. J Morphol. (2006) 267:1177–85. doi: 10.1002/jmor.10464 16830334

[23] Budras K-D Sack WO Röck S Horowitz A Berg R. Chapter 3: pelvic limb In: Budras KD, editor. Anatomy of the horse. 6th ed. Hannover, Germany: Schlütersche (2011)

[24] Beeman GM. The clinical diagnosis of lameness. Comp Cont Educ Pract Vet. (1988) 10:172–9.

[25] Schumacher J Steiger R Schumacher J de Graves F Schramme M Smith R. Effects on analgesia of the distal interphalangeal joint or palmar digital nerves on lameness caused by solar pain in horses. Vet Surg. (2000) 29:54–8. doi: 10.1111/j.1532-950X.2000.00054.x, PMID: 10653495

[26] Hoerdeman M Smith RL Hosgood G. Duration of action of mepivacaine and lidocaine in equine palmar digital perineural blocks in an experimental lameness model. Vet Surg. (2017) 46:986–93. doi: 10.1111/vsu.12689, PMID: 28703891

[27] Adler DMT Dambord P Verwilghen DR. The antimicrobial activity of bupivacaine, lidocaine and mepivacaine against equine pathogens: an investigation of 40 bacterial isolates. The Vet J. (2017) 223:27–31. doi: 10.1016/j.tvjl.2017.05.001, PMID: 28671067

