# Supplementary material for: Computed tomographic evaluation of the proximity of needles placed for perineural anesthesia of the palmar digital nerves to synovial structures in the foot: an ex vivo study
Source: Front Vet Sci. 2024 Jun 4;11:1404331. doi: 10.3389/fvets.2024.1404331 (PMC11183268; doi:10.3389/fvets.2024.1404331)
Supplement: Supplementary file 1 [file Table_1.DOCX]

Supplementary Material

Supplementary Item 1:

|  |  |  | **Adjacent n/total N** | **<5 mm n/total N** | **≥5 and <10 mm n/total N** | **≥10 mm n/total N** |
| --- | --- | --- | --- | --- | --- | --- |
| **NB** | **First distension** | **Proximal**  **Total N=34** | 0 | 0 | 1/34 | 33/34 |
|  |  | **Distal**  **Total N=35** | 0 | 4/35 | 3/35 | 28/35 |
|  | **Second distension** | **Proximal**  **Total N=34** | 0 | 1/34 | 1/34 | 32/34 |
|  |  | **Distal**  **Total N=31** | 0 | 0 | 6/31 | 25/31 |
| **DIPJ** | **First distension** | **Proximal**  **Total N=34** | 1/34 | 0 | 3/34 | 30/34 |
|  |  | **Distal**  **Total N=36** | 1/36 | 4/36 | 19/36 | 12/36 |
|  | **Second distension** | **Proximal**  **Total N=34** | 0 | 4/34 | 8/34 | 22/34 |
|  |  | **Distal**  **Total N=33** | 3/33 | 9/33 | 10/33 | 11/33 |
| **DFTS** | **First distension** | **Proximal**  **Total N=34** | 2/34 | 26/34 | 6/34 | 0 |
|  |  | **Distal**  **Total N=36** | 0 | 24/36 | 8/36 | 4/36 |
|  | **Second distension** | **Proximal**  **Total N=32** | 4/32 | 23/32 | 5/32 | 0 |
|  |  | **Distal**  **Total N=35** | 0 | 25/35 | 8/35 | 2/35 |
|  | **TOTAL (n/420)** |  | 11/420 | 120/420 | 78/420 | 199/420 |

Supplementary Item 1. Overview of number of needle placements categorized as: 1) adjacent needle placement (needle tip adjacent to synovial structure but no contrast leakage), 2) needle tip < 5 mm, 3) between ≥5 - <10 mm and 4) ≥ 10 mm between the needle tip and the synovial structure. The synovial structures were injected with 3 mL, 5 mL and 10 mL 1:1 diluted contrast medium. After each injection (‘First Distension’) needles were inserted just proximal to the palpable edge of the medial and lateral ungular cartilages and were directed distally (‘Distal’). The second needles were placed 1 cm proximal to the first insertion sites ('Proximal'). Subsequently, the same steps were repeated; each synovial structure was injected a second time with 3 mL (NB), 5 mL (DIP) and 10 mL (DFTS) of 1:1 diluted contrast medium to mimic marked synovial distension (‘Second Distension’). N (420) indicates the total number of needle placements in the study. The penetrations (N=12) are not included in this table but can be found in Table 1. NB: navicular bursa; DIPJ: distal interphalangeal joint; DFTS; digital flexor tendon sheath
